# Comparative bench study evaluation of different infant interfaces for non-invasive ventilation

**DOI:** 10.1186/s12890-018-0620-x

**Published:** 2018-04-07

**Authors:** Giorgio Conti, Giorgia Spinazzola, Cesare Gregoretti, Giuliano Ferrone, Andrea Cortegiani, Olimpia Festa, Marco Piastra, Luca Tortorolo, Roberta Costa

**Affiliations:** 1 Intensive Care and Anaesthesia Department and Ventilab, Catholic University of Rome, Policlinico A. Gemelli, Largo Agostino Gemelli 8, 00168 Rome, Italy; 2 Department of Biopathology and Medical Biotechnologies (DIBIMED), Section of Anesthesia, Analgesia, Intensive Care and Emergency. Policlinico Paolo Giaccone, University of Palermo, Via del vespro 129, 90127 Palermo, Italy

**Keywords:** Non invasive ventilation, Bench test, Infant mask, Patient-ventilator interaction, Mechanical ventilation, Acute respiratory failure

## Abstract

**Background:**

To compare, in terms of patient-ventilator interaction and performance, a new nasal mask (Respireo, AirLiquide, FR) with the Endotracheal tube (ET) and a commonly used nasal mask (FPM, Fisher and Paykel, NZ) for delivering Pressure Support Ventilation (PSV) in an infant model of Acute Respiratory Failure (ARF).

**Methods:**

An active test lung (ASL 5000) connected to an infant mannequin through 3 different interfaces (Respireo, ET and FPM), was ventilated with a standard ICU ventilator set in PSV. The test lung was set to simulate a 5.5 kg infant with ARF, breathing at 50 and 60 breaths/min). Non-invasive ventilation (NIV) mode was not used and the leaks were nearly zero.

**Results:**

The ET showed the shortest inspiratory trigger delay and pressurization time compared to FPM and Respireo (*p* < 0.01). At each respiratory rate tested, the FPM showed the shortest Expiratory trigger delay compared to ET and Respireo (*p* < 0.01). The Respireo presented a lower value of Inspiratory pressure–time product and trigger pressure drop than ET (*p* < 0.01), while no significant difference was found in terms of pressure-time product at 300 and 500 ms. During all tests, compared with the FPM, ET showed a significantly higher tidal volume (V_T_) delivered (*p* < 0.01), while Respireo showed a trend toward an increase of tidal volume delivered compared with FPM.

**Conclusions:**

The ET showed a better patient-ventilator interaction and performance compared to both the nasal masks. Despite the higher internal volume, Respireo showed a trend toward an increase of the delivered tidal volume; globally, its efficiency in terms of patient-ventilator interaction was comparable to the FPM, which is the infant NIV mask characterized by the smaller internal volume among the (few) models on the market.

## Background

The role of Non-Invasive Ventilation (NIV) in children with acute respiratory failure (ARF) treated in the Pediatric Intensive Care Unit (PICU) is well established [1–7].

During NIV, ventilator modes, settings and interfaces may deeply affect patient-ventilator interaction. Pressure Support Ventilation (PSV) still remains the mode most commonly used in PICU during NIV, although Neurally Adjusted Ventilatory Assist (NAVA) has been recently proposed to improve patient-ventilator synchrony in infants [8, 9]. Nevertheless, NAVA requires the placement of an indwelling catheter making its use more invasive and expensive [8–10]. As a matter of fact, the use of a comfortable and well-fitted interface, as well as an appropriate ventilator mode and setting are both important factors to optimize patient-ventilator interaction and increase patient’s compliance during NIV [6, 11].

NIV is usually delivered with different interfaces, such as face and nasal masks or helmets. However, only few pediatric interfaces are present on the market and, more often than never, their sub-optimal design can deeply affect patient-ventilator synchrony, compared to the benchmark, represented by the endotracheal tube. In a recent study on a pediatric model breathing at high respiratory rates, the helmet demonstrated the worst patient-ventilator interaction, suggesting that the face mask should be considered the first choice for delivering NIV in babies [6].

Nevertheless, considering that infants are usually nose breathers, the nasal mask is largely employed in this patient population [12]. So far, no study has investigated the role of different nasal interfaces on patient-ventilator interaction in infants, even though nasal masks may have different internal volumes and may behave differently in various clinical settings.

We hypothesized that, compared to the ET, considered as the benchmark, different nasal masks with specific features in terms of internal volume and dead space could perform differently in terms of patient- ventilator synchrony. In order to test this hypothesis, a comparative bench study using an active lung simulator connected to a mannequin was designed to determine whether different interfaces and ventilator settings might influence patient-ventilator interaction in an infant model of restrictive respiratory failure.

## Methods

This study was performed at the Respiratory Mechanics Laboratory (Ventil@b) of the Catholic University of Rome, Italy. A Laerdal Resusci Baby mannequin (Laerdal Medical, Stavanger, Norway) has been chosen for this study, being the most widely used resuscitation mannequin and the most realistic for the purposes of our bench study [13–16].

We connected the artificial airway of the mannequin with an active test lung (ASL 5000, Ingmar Medical, Pittsburgh, Pennsylvania) in order to test three different interfaces: the endotracheal tube (ET, size ID 4 mm, Covidien, Mansfield, Massachusetts), a new infant nasal mask (Respireo, extrasmall size, AirLiquide, FR) and a commonly used infant nasal mask (FPM, Infant Nasal Mask, large size, Fisher and Paykel, NZ) [17–19]. The two masks differ for shape and design characteristic, FPM presenting two parallel connections with a complete separation between inspiratory and expiratory limbs, while Respireo is characterized by a single limb connected to the Y piece with a flexible tube able to rotate at 360°.

A standard intensive care unit (ICU) ventilator wad used to ventilate the Resusci Baby mannequin (Servo I, Maquet, Sweden) [20, 21] in neonatal PSV mode, without using the air leak compensation software, since air leaks were eliminated during NIV by sealing the masks to the mannequin’s face. The ET and the masks were connected to the ventilator using a standard double limbs neonatal circuit. The mouth of the mannequin was filled and closed to reduce the dead space. Pressure Support (PS) and Positive End Expiratory Pressure (PEEP) were set at13cmH_2_O and 5 cmH_2_O, respectively. The inspiratory flow trigger was set at 1.5 L/min and optimized to the lowest level, to avoid auto-trigger.

Inspiratory trigger, pressurization time (Time_press_) and expiratory trigger threshold (Tr_exp_) were set to optimize patient-ventilator interaction and maintained constant throughout the trials. The test lung was set to simulate a 5.5 Kg BW infant, with a restrictive condition simulating a mild Acute Respiratory Distress Syndrome (ARDS). Compliance was set at 0.8 ml/cmH_2_O/kg, respiratory system resistances at 25 cmH_2_O/L/s and inspiratory muscle pressure (Pmus) at 12 cmH_2_O. Respiratory Rate (RR) was set at 50 and 60 breaths/min. Each test condition lasted 20 min, and the last 5 min of each trial were recorded for analysis.

### Data acquisition and analysis

**Fig. 1:**
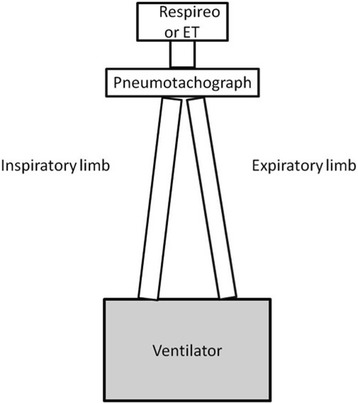
Schematic representation of a bench study setting with the new infant nasal mask (Respireo) or the endotracheal tube (ET)

Air flow (V′) was measured with a pneumotachograph (Fleish No.1, Metabo, Epalinges, Switzerland), while airway pressure (P_aw_) was measured by a pressure transducer with a differential pressure of ±100 cmH_2_O (Digima Clic-1, ICULab system; KleisTek Engineering, Bari, Italy), placed distally from the pneumotachograph. When the mannequin was ventilated through the ET or the Respireo, the pneumotachograph and the pressure transducer were positioned at the Y-connection of the ventilator circuit (Fig. 1). In the FPM, Flow and P_aw_ were not measured because of the mask design, which determines a complete separation of inspiratory and expiratory limbs, not allowing the correct positioning of an external pneumotachograph. All the signals were acquired, amplified, filtered, and digitized at 100 Hz, then recorded on a dedicated personal computer and analyzed through specific software (ICULab 2.7; KleisTek). Ventilator inspiratory and expiratory time (mechanical T_I_ and mechanical T_E_, respectively), and ventilator rate of cycling were all determined on the flow tracing. The inspiratory duty cycle (mechanical T_I_/T_tot_) was calculated as the ratio between mechanical T_I_and the total mechanical breath duration (T_tot_). Airflow (V′) and tidal volume (V_T_) delivered to the simulator, airway opening pressure (Paw), and inspiratory muscles effort were displayed online on the computer screen. The signals obtained with the ASL were transmitted to a PC host via 10/100MBit Ethernet, sampled and processed in real time by means of specific software (Lab View, Ingmar Medical). The signals obtained with the ASL were integrated with the signals from the ICULab system by using a specific application of the ICULab (ICULab 2.7, KleisTek). The numerical integration of flow over time determined the mechanical tidal volume (mechanical V_T_). The amount of tidal volume delivered to the simulator during its active inspiration (ie, the neural tidal volume, V_T_) was calculated as the volume generated from the onset of inspiratory muscle effort negative deflection to its nadir. Interfaces performance was evaluated using the following parameters [20–22]:

1. Trigger pressure drop (Swing_trigger_), defined as the pressure swing generated by the simulator inspiratory effort in the airway during the triggering phase;
2. Inspiratory pressure–time product (PTP_trigger_), defined as the area under the Paw curve relative to the time between the onset of inspiratory effort and the start of mechanical assistance;
3. Pressure-time product at 300 ms (PTP_300_) defined as the integration of Paw over time during the first 300 msec and representing the speediness of the ventilator in reaching the preset level of pressure support;
4. Pressure-time product at 500 ms (PTP_500_), defined as the integral Paw area over insufflation time from the simulated effort onset, representing the ventilator capability of maintaining the pressurization;
5. PTP_500_ index, expressed as a percentage of the ideal PTP, which is unattainable because it would imply a trigger pressure drop and an instantaneous pressurization of the ventilator.

**Fig. 2:**
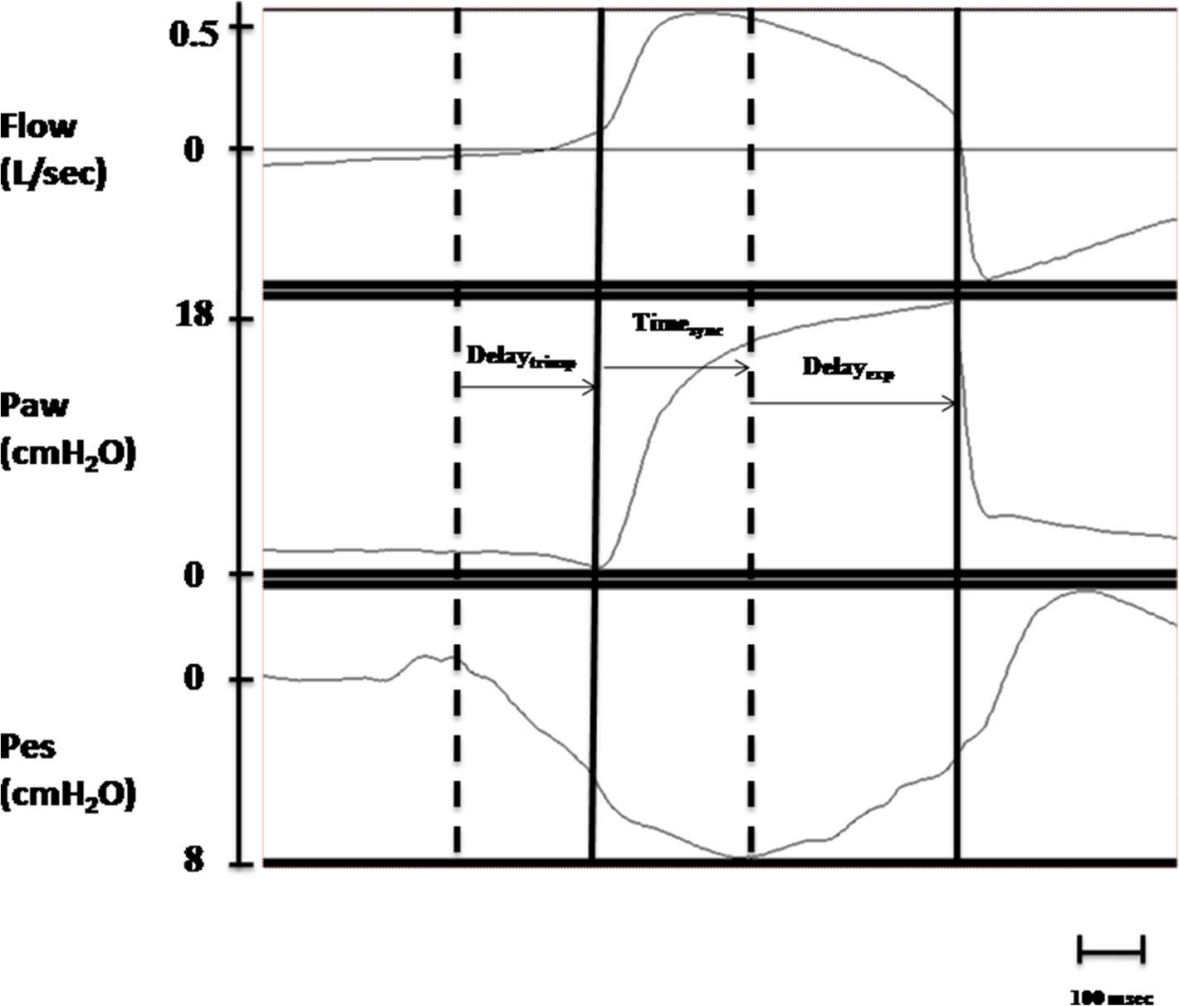
Example from a real patient tracing (from our database) of patient-ventilator interaction measurements during NIV. From the top to the bottom: Flow (V′), Airway pressure (Paw) and Esophageal pressure (Pes). Delay_trinsp_: between the first dotted line and the first black line is the delay between the onset of patient inspiration and the start of the mechanical assistance. Delay_trexp_: between the second dotted line and the second black line is the delay between the end of patient inspiration and the end of the mechanical insufflation. Time_sync_: between the first black line and the second dotted line is the time during which the patient and the ventilator are in phase

Patient–ventilator interaction (Fig. 2) was evaluated by determining:

1. Pressurization time (Time_press_), defined as the time necessary to achieve the pre-set level of pressure support from the baseline value;
2. Inspiratory trigger delay (Delay_trinsp_), calculated as the time lag between the onset of inspiratory muscle effort negative swing and the start of the ventilator support (i.e., P_aw_positive deflection);
3. Expiratory trigger delay (Delay_trexp_), assessed as the delay between the end of the inspiratory effort and the end of the mechanical insufflations (i.e., flow deflection);
4. Time of synchrony (Time_sync_), defined as the time during which inspiratory muscle effort and Paw are in phase (ideally 100%);
5. SimulatorV_T_/mechanicalV_T_, intended as the percentage of V_T_ delivered during inspiratory muscle effort negative deflection;
6. Wasted efforts, defined as ineffective inspiratory efforts, not assisted by the ventilator;
7. Auto-triggering, namely a mechanical insufflation in absence of inspiratory effort.

### Statistical analysis

All data are expressed as mean ± SD. All variables were compared with each interface used. All variables were compared by using a non-parametric Kruskal-Wallis test for analysis of variance (ANOVA) on ranks. Pairwise comparisons were done with the Dunn’s multiple comparison test. The Mantel-Haenszel extended chi-square test was used. *P* value < 0.05 was considered statistically significant.

## Results

**Fig. 3:**
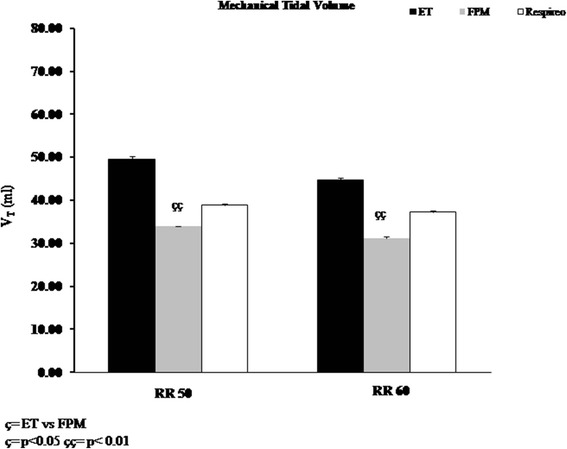
Mechanical Tidal Volume (V_T_) with the endotracheal tube (ET) (black column), the Fisher and Paykel infant nasal mask (FPM) (gray column) and the new infant nasal mask (Respireo) (white column) at two different Respiratory Rates (RR 50 and 60 breath/min). The V_T_ can be expressed also in ml per kg as follows: ET 9.1 ml/kg, FPM 6 ml/kg, Respireo 7.1 ml/kg (at RR 50); ET 8.2 ml/kg, FPM 5.8 ml/kg, Respireo 6.9 ml/kg (at RR 60)

During all study conditions, the V_T_ delivered to the mannequin was significantly higher with the ET than with the FPM (*p* < 0.01). No significant differences were found in terms of V_T_ during Respireo NIV compared to the other two settings, although this mask showed a not trend toward an increase of the delivered V_T_ compared to the FPM (Fig. 3).

At RR 50 the ET showed significantly shorter Delay_trinsp_ and Time_press_compared to the Respireo (*p* < 0.05), while no significant differences were observed between the two masks. At RR 60 no difference was observed in terms of Delay_trinsp_ between the three interfaces.

**Fig. 4:**
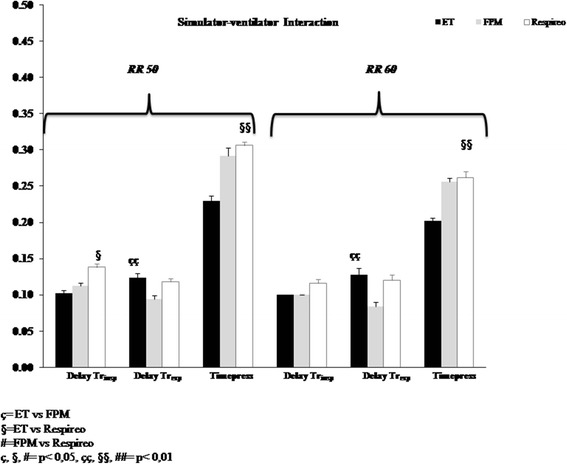
Inspiratory trigger delay (Delay_trinsp_), Pressurization Time (Time_press_) and Expiratory Trigger delay (Dealy_trexp_) with the endotracheal tube (ET) (black column), the Fisher and Paykel infant nasal mask (FPM) (gray column) and the new infant nasal mask (Respireo) (white column) at two different Respiratory Rates (RR 50 and 60 breath/min)

At both RR tested, the FPM showed a significantly shorter Delay_trexp_ compared both to the ET and the Respireo (*p* < 0.01) (Fig. 4).

**Fig. 5:**
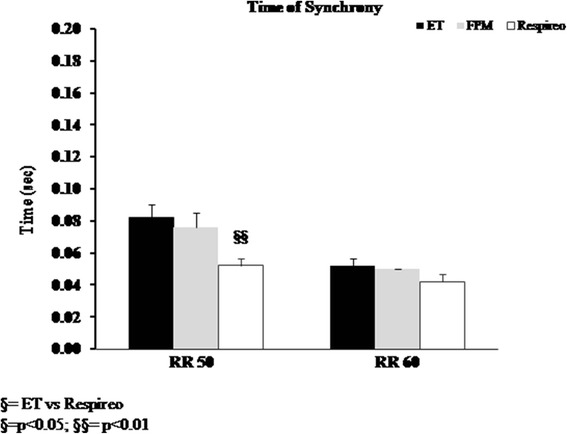
Time of synchrony with the endotracheal tube (ET) (black column), the Fisher and Paykel infant nasal mask (FPM) (gray column) and the new infant nasal mask (Respireo) (white column) at two different Respiratory Rates (RR 50 and 60 breath/min)

At RR60 Time of Synchrony (Time_sync_) did not show significant differences between all the interfaces, while at RR50 the Respireo, but not the FPM, showed a significantly shorter Time_sync_ compared to the ET (*p* < 0.01) (Fig. 5).

The performance analysis was conducted only between the ET and the Respireo, as the FPM design did not allow positioning an external pneumotacograph. At both RR, the Respireo showed a significantly shorter Swing_trigger_ and PTP_trigger_ compared to the ET (*p* < 0.01). No significant difference was found between the Respireo and the ET in terms of PTP_300_ at both RR tested. Finally, at RR 60 the Respireo showed a better PTP_500_index compared to the ET (*p* < 0.05) (Table 1).

**Table 1:** Interfaces performance

RR 50	ET	Respireo	*P*	RR 60	ET	Respireo	*p*
Swing_trigger_ (cmH2O)	1.82 ± 0.1	0.83 ± 0.08	< 0.001		2.75 ± 0.12	0.78 ± 0.09	< 0.001
PTP_trigger_ (cmH2O/s)	0.09 ± 0.01	0.06 ± 0.01	< 0.001		0.14 ± 0.01	0.05 ± 0.02	< 0.001
PTP_300_ (cmH2O/s)	1.8 ± 0.11	1.61 ± 0.19	NS		1.7 ± 0.1	1.7 ± 0.18	NS
PTP_500_index (%)	55%	56%	NS		48%	50%	< 0.05

Performance evaluation of the endotracheal tube (ET) and the new infant nasal mask (Respireo) in terms of Trigger pressure drop (Swing_trigger_), Inspiratory pressure–time product (PTP _trigger_), Pressure-Time Product at 300(PTP_300_), and PTP_500_ index at two respiratory rates (RR 50 and RR 60 breaths/min)

Values are mean ± SD

## Discussion

To the best of our knowledge, this is the first study aimed at evaluating different NIV interfaces in a simulated infant restrictive model. The main results of this bench study can be summarized as follows:

1. At RR 50 the ET showed a better patient-ventilator interaction in terms of Delay_trinsp_ and Time_press_ compared to the nasal masks tested. At RR 60, no difference was observed in terms of Delay_trinsp_ between the three interfaces. The Respireo showed better Swing_trigger_ and PTP_trigger_ compared to the ET at both RR.
2. The V_T_ delivered to the mannequin was between 6 and 8 ml/Kg, although, during ET, V_T_ showed a trend toward an increase compared during Respireo NIV and it was significantly higher than during FPM NIV. No differences were found between ET and Respireo and between Respireo and FPM.
3. No significant differences were observed in terms of PTP_300_ and PTP_500_ between the Respireo and the ET. Nevertheless, at RR 60 the Respireo showed a significantly better performance in terms of PTP _500_index compared to the ET.

Despite the ET represents the standard of care for the treatment of ARF in infants, there is an increasing evidence of physicians trying to avoid intubation or extubate their patients and continue the ventilator assistance on NIV [23].

In the last years, many efforts have been made to improve the interfaces. This has involved interface physical characteristics, materials and design. Neonates and infants are preferentially nose breathers and the choice of the interface is determined both by the age and by the type of ventilator support. During NIV the likelihood of leaks, with subsequent patient-ventilator asynchrony is higher than during nasal Continuous Positive Airway Pressure (CPAP) [24].

In order to understand this issue, it is important to note that in PICU, CPAP is delivered through “leaking systems”, where intentional air leaks are an intrinsic feature of the CPAP system. In the same way, NIV can be administered using intentional leaks ventilators, namely ventilators that are coupled with masks provided with embedded “exhalation holes”, also named as vented masks. Conversely, in our bench study, NIV was delivered by an high pressure ICU ventilator with active valves adopting a double circuit, without any intentional leak. For these reasons we used a non-vented mask.

We chose to use PSV to compare nasal masks with the ET, where mechanical ventilation is provided with high pressure ICU ventilator. We used an infant mannequin [25] and developed a system that allowed to avoid air leaks during both mask and ET ventilation, although we are aware that non intentional leaks are routinely observed both during invasive and non-invasive ventilation in infants [26, 27]. In this study we wanted to have an accurate estimation of flow and pressure curves as well as of the delivered V_T_ during simulated positive pressure ventilation. In addition, other studies where leaks were allowed tested the efficiency of face masks in the resuscitation of newborn infants [26, 28], measuring the expired tidal volumes during bag and mask ventilation or NIV similar to our setting with a flow sensor (pneumotachograph) placed between the Y connection of the circuit and the interface [29–31].Our results demonstrated that the ET, as expected, showed an overall better patient-ventilator interaction compared to the nasal masks at both RR tested. Interestingly, the Respireo showed a better Swing_trigger_ and PTP_trigger_ compared to the ET at both RR. This result can be explained considering the higher inspiratory resistance generated by the ET compared to the Respireo mask, that determines a deeper Swing trigger and thus, an higher PTP trigger.

In addition, compared to the FPM, the Respireo showed a trend toward an increase of the delivered V_T_ at both RR. This result may be explained considering, on one side, that Respireo has a double internal volume than FPM and on the other that FPM, for its specific design, generates a complete separation of the inspiratory and expiratory limbs, thus increasing inspiratory resistances with consequent generation of lower V_T_ for a comparable level of transpulmonary pressure. It can also be speculated that the Respireo lower resistance, compared to the ET, was responsible of a better performance in terms of PTP _500_ index at RR 60.

Our study has several limitations that need to be discussed. The major limitation is that it is a bench study conducted on an active lung simulator breathing with a repetitive respiratory rhythm that does not fully represent the clinical behavior of an infant receiving NIV. Moreover, during a bench study, the interfaces are evaluated in “optimal” conditions (i.e. without air leaks or secretions) to obtain a pure performance evaluation. For all these reasons, our results need to be confirmed by clinical studies assessing the effectiveness of the masks in ventilating infants in different conditions and evaluating their performance in response to the variability of a real clinical scenario.

Unfortunately, it is technically and ethically impossible to perform a direct comparison between different interfaces, especially when an ET is included in the same (pediatric) patient. Moreover, despite the mannequin used is considered one of the best devices for simulation, the anatomy of the upper airways is not perfectly representative of the human infant ones. In details, the nostrils are probably more resistive than the “in vivo” nostrils and the rhino- and oro-pharynx are larger than the “in vivo” ones. These differences increase both the resistive work (nostrils), and the dead space, making the “in vitro” study setting adopted in this evaluation a worst scenario than the “in vivo” conditions.

## Conclusions

We have developed an active model for assessing the delivery of invasive and non-invasive ventilation in infants. With this model, the ET showed a better patient-ventilator interaction and performance compared to the nasal masks. Respireo was superior to the FPM in terms of delivered V_T_ at both RR; at the higher RR both masks showed similar results, despite the double internal volume of Respireo. Respireo showed a better Swing_trigger_ and PTP_trigger_ compared to the ET, while in terms of pressurization and PTP_300_ and PTP_500_ the results were similar. Globally, the Respireo performance was comparable and sometime superior to the FPM, which is the infant NIV mask characterized by the smaller internal volume among the (few) models on the market.

## Abbreviations

ARDS: Acute respiratory distress syndrome
ARF: Acute respiratory failure
Delay_trexp_: Expiratory trigger delay
Delay_trinsp_: Inspiratory trigger delay
ET: Endotracheal tube
ICU: Intensive care unit
NAVA: Neurally Adjusted Ventilatory Assist
NIV: Noninvasive ventilation
P_aW_: Airway pressure
PEEP: Positive end expiratory pressure
Pmus: Inspiratory muscle pressure
PS: Pressure support
PSV: Pressure support ventilation
PTP_500_: Pressure-time product at 500 ms
PTP_trigger_: Inspiratory pressure–time product
RR: Respiratory Rate
Swing_trigger_: Trigger pressure drop
T_E_: Expiratory time
T_I_: Inspiratory time
T_I_/T_tot_: Inspiratory duty cycle
Time_press_: Pressurization ramp
Time_sync_: Time of synchrony
Tr_exp_: Expiratory trigger threshold
T_tot_: Total mechanical breath duration
V′: Air flow
